# Genome of the parasitoid wasp *Cotesia chilonis* sheds light on amino acid resource exploitation

**DOI:** 10.1186/s12915-022-01313-3

**Published:** 2022-05-24

**Authors:** Xinhai Ye, Shijiao Xiong, Ziwen Teng, Yi Yang, Jiale Wang, Kaili Yu, Huizi Wu, Yang Mei, Cheng Xue, Zhichao Yan, Chuanlin Yin, Fang Wang, Hongwei Yao, Qi Fang, Qisheng Song, Gongyin Ye, Fei Li

**Affiliations:** 1 State Key Laboratory of Rice Biology and Ministry of Agricultural and Rural Affairs Key Laboratory of Molecular Biology of Crop Pathogens and Insects, Institute of Insect Sciences, Zhejiang University, Hangzhou, China; 2 Shanghai Institute for Advanced Study, Zhejiang University, Shanghai, China; 3 College of Computer Science and Technology, Zhejiang University, Hangzhou, China; 4 Department of Biology, University of Rochester, Rochester, NY USA; 5 Division of Plant Sciences, College of Agriculture, Food and Natural Resources, University of Missouri, Columbia, MO USA

**Keywords:** Amino acid synthesis, Trait loss, Nutrition exploitation, Genome sequencing, Parasitoid wasps, *Cotesia chilonis*

## Abstract

**Background:**

A fundamental feature of parasitism is the nutritional exploitation of host organisms by their parasites. Parasitoid wasps lay eggs on arthropod hosts, exploiting them for nutrition to support larval development by using diverse effectors aimed at regulating host metabolism. However, the genetic components and molecular mechanisms at the basis of such exploitation, especially the utilization of host amino acid resources, remain largely unknown. To address this question, here, we present a chromosome-level genome assembly of the parasitoid wasp *Cotesia chilonis* and reconstruct its amino acid biosynthetic pathway.

**Results:**

Analyses of the amino acid synthetic pathway indicate that *C. chilonis* lost the ability to synthesize ten amino acids, which was confirmed by feeding experiments with amino acid-depleted media. Of the ten pathways, nine are known to have been lost in the common ancestor of animals. We find that the ability to synthesize arginine was also lost in *C. chilonis* because of the absence of two key genes in the arginine synthesis pathway. Further analyses of the genomes of 72 arthropods species show that the loss of arginine synthesis is common in arthropods. Metabolomic analyses by UPLC-MS/MS reveal that the temporal concentrations of arginine, serine, tyrosine, and alanine are significantly higher in host (*Chilo suppressalis*) hemolymph at 3 days after parasitism, whereas the temporal levels of 5-hydroxylysine, glutamic acid, methionine, and lysine are significantly lower. We sequence the transcriptomes of a parasitized host and non-parasitized control. Differential gene expression analyses using these transcriptomes indicate that parasitoid wasps inhibit amino acid utilization and activate protein degradation in the host, likely resulting in the increase of amino acid content in host hemolymph.

**Conclusions:**

We sequenced the genome of a parasitoid wasp, *C. chilonis*, and revealed the features of trait loss in amino acid biosynthesis. Our work provides new insights into amino acid exploitation by parasitoid wasps, and this knowledge can specifically be used to design parasitoid artificial diets that potentially benefit mass rearing of parasitoids for pest control.

**Supplementary Information:**

The online version contains supplementary material available at 10.1186/s12915-022-01313-3.

## Background

Parasites are ubiquitous in nature, and nutrition exploitation is one of the most important aspects of parasitism [1]. Understanding the genetic basis of nutrition exploitation is a fundamental goal in the study of parasites and could provide insight into new methods for pest control. Parasitoid wasps are one of the most successful parasitic groups with an amazing diversity of species, and they have evolved a number of strategies to manipulate their hosts to ensure the success of parasitism. Female parasitoid wasps attack arthropod hosts and lay their eggs upon or within them, where the offspring feed and develop, eventually causing host death [2–4]. Some parasitoid wasps are important biological control agents in integrated pest management [5].

Parasitoid wasps provide a promising model to study parasite-host interaction and nutrition exploitation by parasites. Parasitoid effectors, such as venom proteins, polydnaviruses (PDVs), and parasitoid cells (teratocytes), produced by female wasps or larvae are important weapons that are used to regulate host physiological processes for the purpose of protecting offspring survival in the host [2, 6, 7]. In addition to inhibiting host immunity and altering host development, parasitoid wasp effectors manipulate the host into producing a nutritionally favorable environment for development [1, 8]. For instance, parasitoid venoms induce an increase in the concentration of lipids [9, 10]. Parasitism by *Nasonia vitripennis* dramatically alters the gene expression and the levels of sugars, lipids, and amino acids in the host [8, 11]. Parasitism-induced nutrition changes have also been found in the other parasitoid-host systems. For example, *Aphidius ervi* parasitism increases the concentrations of nutritional components such as proteins, acylglycerols, and total free amino acids in the host hemolymph [12, 13]; *Lysiphlebia japonica* increases fatty acid content in the early stage of parasitism [14]; and *Pteromalus puparum* parasitism induces a significant change in lipid levels in the fat body and hemolymph of the host [15]. These studies revealed that nutritional components change during parasitism. However, the molecular mechanisms underlying nutrition exploitation remain largely unknown.

Loss of the ability to biosynthesize nutrients has been discovered in several insects. For example, because they can obtain the nutrition from their endosymbionts, some hemipteran insects, e.g., aphids, planthoppers, and mealybugs, have lost genes in the amino acid biosynthetic pathways [16–20]. Since the carnivorous larvae of parasitoid wasps feed on protein- and lipid-rich food resources from their hosts, gene loss in some biosynthetic pathways is frequently observed in parasitoid wasps. For example, previous studies have shown that most parasitoid wasps lack lipogenesis, but they did not find the extensive losses of genes involved in lipogenesis [21–23]. In many insects, feeding assays have demonstrated that nine or ten amino acids are required in the diet, and if the amount of one of these amino acids is insufficient, the insect fails to develop [24]. Thus, these amino acids are known as essential amino acids. The biosynthesis pathways of nine of these amino acids (leucine, isoleucine, valine, methionine, histidine, lysine, threonine, tryptophan, and phenylalanine) were lost in the stem lineage of animals. Thus, they are essential amino acids for all animals (ASL-AA) [25]. The biosynthetic pathway of arginine via the urea cycle is retained in many insects, but most of them also need a dietary supply of arginine, which is probably due to the low synthesis efficiency [24]. The genetic basis of amino acid biosynthesis in parasitoid wasps, especially possible gene loss in the amino acid biosynthetic pathways, is less understood at present.

**Fig. 1:**
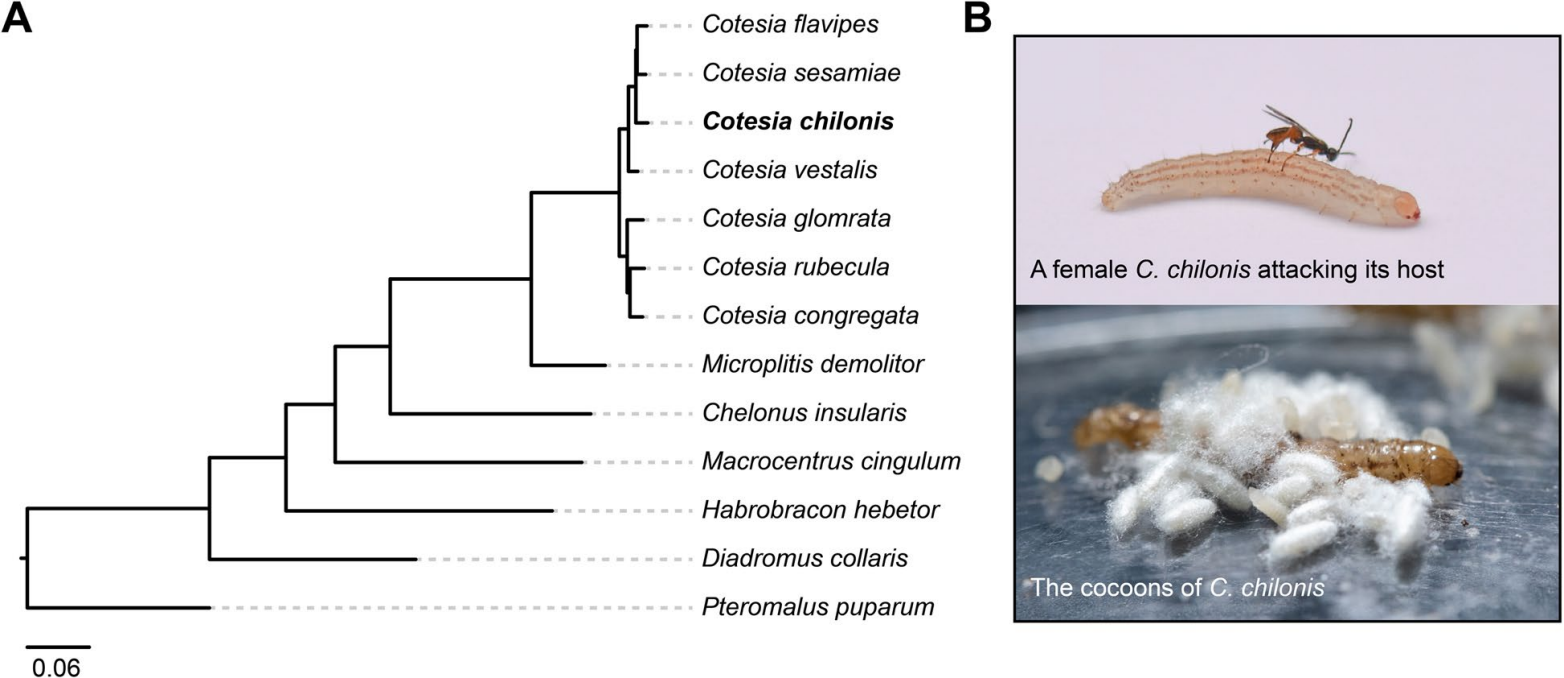
Phylogenetic relationship among *C. chilonis* and other 12 parasitoid wasps. **A** A phylogenetic tree of *C. chilonis* with other parasitoid wasps based on 2179 single-copy orthologous genes by IQ-TREE maximum likelihood method and 1000 bootstrap replicates. All nodes received 100% bootstrap support. **B** A female *C. chilonis* attacking its host *C. suppressalis* and the cocoons of *C. chilonis*

To fill this gap in knowledge, we conducted genome sequencing of the Bracovirus (a family of PDVs)-carrying endoparasitoid wasp *Cotesia chilonis* (Hymenoptera: Braconidae), which is a natural enemy of the notorious insect pest *Chilo suppressalis* (rice stem borer) and is widely used as a biological control agent (Fig. 1B) [26, 27]. We present genome-based evidence for the loss of the biosynthesis pathways of ten amino acids (ASL-AA and arginine) in *C. chilonis*, followed by experimental validation using feeding assays with amino acid-depleted media. Next, we use ultra-performance liquid chromatography tandem mass spectrometry (UPLC-MS/MS) to demonstrate that parasitism temporally increases or decreases the contents of some amino acids in host hemolymph. Transcriptomic analyses suggest that the increase in amino acid content in host hemolymph is likely induced by the counterbalance of decreased host amino acid utilization and increased host protein degradation.

## Results

### Chromosomal level genome of *C. chilonis*

We combined Illumina short reads, PacBio long reads, and Hi-C chromosomal contact information to generate a chromosome-level reference assembly of the parasitoid wasp *C. chilonis*. The final assembly was 189 Mb. The scaffolds were anchored to 10 chromosomes with a superscaffold N50 of 20.4 Mb (Table 1). Benchmarking Universal Single-Copy Ortholog (BUSCO) assessment [28] indicated that more than 97% of genes (1319) were captured, with only 26 missing genes, and 12 fragmented genes, suggesting the high quality of this genome assembly (Additional file 1: Tables S1-S3 and Additional file 2: Fig. S1).

**Table 1:** Statistics for the *C. chilonis* chromosomal genome assembly and gene annotations

Assembled genome	
Total length (contigs)	189,230,571 bp
N50 length (contigs)	1,215,404 bp
Total length (scaffolds)	189,587,396 bp
N50 length (scaffolds)	20,361,094 bp
Number of assembled chromosomes	10
Largest chromosome	27,246,087 bp
Hi-C anchoring ratio	91.93%
BUSCO analysis	
Complete	1319
Duplicated	10
Fragmented	12
Missing	26
Genome features	
Repeat (%)	36.18
GC content (%)	30.36
Protein coding genes	14,142

We annotated 353,649 repeat sequences in a total of 68 Mb, which constitutes 36% of the genome (Additional file 1: Table S4). Next, by integrating three aspects of evidence from de novo prediction, homology alignments, and gene expression, the Optimized Maker-Based Insect Genome Annotation (OMIGA) pipeline [29] identified 14,142 protein-coding genes in the *C. chilonis* genome (Table 1). A total of 9617 genes were supported by Swiss-Prot, and 10,639 genes were assigned annotations in the Kyoto Encyclopedia of Genes and Genomes (KEGG).

The phylogenetic relationships between *C. chilonis* and the other 12 parasitoid wasps were determined using a set of 2179 single-copy orthologous genes with *Pteromalus puparum* (Chalcidoidea, Pteromalidae) as the outgroup. As expected, phylogenetic analysis confirmed that *Diadromus collaris* (Ichneumonoidea, Ichneumonidae) was a sister group to the other 11 braconid wasps (Ichneumonoidea, Braconidae) (Fig. 1A). The seven *Cotesia* species were clustered into two clades: clade 1 included *C. glomrata*, *C. rubecula*, and *C. congregate*, while clade 2 included *C. flavipes*, *C. sesamiae*, *C. vestalis*, and *C. chilonis*. In clade 2, *C. chilonis* is a sister group to the lineage containing *C. flavipes* and *C. sesamiae* (Fig. 1A). This phylogeny is overall consistent with previous studies on hymenopterans [30] and illustrates the phylogenetic relationship of *C. chilonis* to other *Cotesia* species for the first time.

### Gene loss in the biosynthetic pathways of ten amino acids in *C. chilonis*

Though much efforts have been devoted to elucidating the immune manipulation of hosts by parasitoid wasps, less is known about the interactions between the host and parasitoid in terms of nutrition [1]. Because the genome of the host *C. suppressalis* is available [31], *C. chilonis*-*C. suppressalis* is an excellent model system to investigate the genetic basis of the utilization of host nutrients by parasitoid wasps. Thus, we used this parasitoid-host model to investigate the molecular mechanism of host amino acid exploitation by parasitoid wasps.

**Fig. 2:**
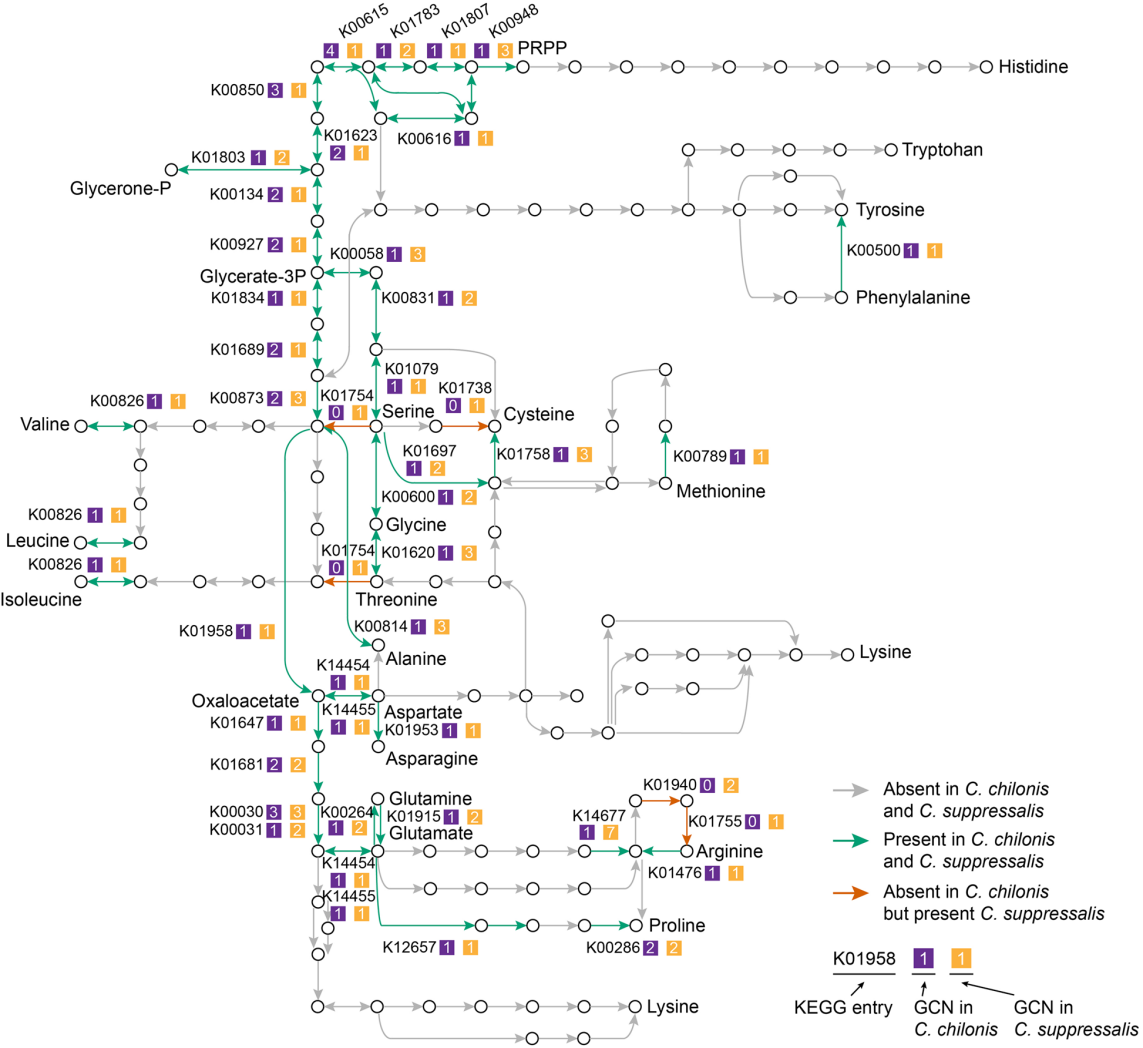
The amino acid biosynthetic pathway in the parasitoid wasp *C. chilonis* and its host *C. suppressalis*. The amino acid biosynthetic pathways were redrawn from the KEGG pathway, map01230. The pathway genes in gray are lost in both genomes of *C. chilonis* and *C. suppressalis*. The pathway genes in green are present in both genomes of *C. chilonis* and *C. suppressalis*. The pathway genes in orange are present in the genome of *C. suppressalis* but are lost in the genome of *C. chilonis*. The gene losses in the amino acid biosynthetic pathway of *C. chilonis* have disrupted the biosynthesis of ten amino acids (ASL-AA, Arg). However, the gene losses in the amino acid biosynthetic pathway of *C. suppressalis* have disrupted the biosynthesis of nine amino acids (ASL-AA), and the biosynthesis of these nine amino acids was thought to have been lost in the common ancestor of animals [25]. Gene copy number (GCN) of each pathway ortholog in *C. chilonis* and *C. suppressalis* was shown

First, we asked what amino acids are essential for *C. chilonis* during its life cycle. To this end, we reconstructed the amino acid biosynthetic pathways of *C. chilonis* and examined the pathway disruptions and ability to synthesize different amino acids. KEGG modules (or sub-pathways) for amino acid biosynthesis were used [32]. We define pathway disruption as the loss of one or more genes required for amino acid synthesis in the pathway. The loss of the ability to synthesize an amino acid occurs when all synthetic pathways for this amino acid are disrupted in such a way that there is no complete path for synthesis based on the currently known pathways. In total, we found 28 pathway disruptions in *C. chilonis*, and these disrupted pathways are related to the synthesis of 12 amino acids, including ASL-AA, cysteine, arginine, and tyrosine (Additional file 1: Table S5). However, the ability to synthesize cysteine and tyrosine appears to have been retained because of redundancy in pathways for these two amino acids. For cysteine, although the pathways from 3-phosphoserine to cysteine and from *O*-acetyl-l-serine to cysteine were disrupted by gene losses, cysteine can also be converted from l-cystathionine by cystathionine gamma-lyase (K01758) in M00338 (Fig. 2 and Additional file 1: Table S5). For tyrosine, it can be synthesized from phenylalanine by phenylalanine-4-hydroxylase (K00500) (Fig. 2 and Additional file 1: Table S5). Therefore, from a genomic point of view, the gene losses in *C. chilonis* have disrupted the biosynthesis of ten amino acids (ASL-AA and arginine).

To test if there is nutrition compensation between *C. chilonis* and its host *C. suppressalis*, we next conducted a comparative pathway analysis between them. The overall pathway disruption patterns are largely similar between the parasitoid *C. chilonis* and its host. Only four amino acid synthetic pathway genes lost in the parasitoid were present in the host genome (Fig. 2). Among them, argininosuccinate synthase (K01940) and argininosuccinate lyase (K01755) are in the arginine biosynthetic pathway, cysteine synthase (K01738) is from the cysteine biosynthetic pathway, and threonine dehydratase (K01754) is from the biosynthetic pathway of valine, leucine, and isoleucine (Fig. 2). Because of the existence of argininosuccinate synthase and argininosuccinate lyase, the arginine synthetic pathway is complete in *C. suppressalis*. However, the synthesis of cysteine and valine, leucine, and isoleucine remain disrupted despite the existence of cysteine synthase and threonine dehydratase, respectively, because of the losses of other key pathway genes. Taking all this evidence together, the biosynthesis of nine amino acids (ASL-AA) is disrupted in the host *C. suppressalis*.

To place the pathway repertoire changes between *C. chilonis* and *C. suppressalis* in an evolutionary standpoint, we further examined the amino acid synthesis gene repertoires of 72 additional arthropods, including six Hemipterans, 21 Hymenopterans, eight Lepidopterans, 23 Dipterans, four Coleopterans, one Phthirapterans, one Blattodeans, one Collembolans, two Crustaceans, and five chelicerate species. The threonine dehydratase gene was found in most of these arthropods and only lost in 15 species from Hymenoptera (7), Hemiptera (1), Phthiraptera (1), Malacostraca (1), Collembola (1), and Arachnida (4) (Additional file 1: Table S6). This suggested that several independent gene loss events have occurred during Arthropoda evolution. In Hymenoptera, the threonine dehydratase gene was lost in all three wasps in the infraorder Parasitoida (*Microplitis demolitor*, *Nasonia vitripennis*, *Ceratosolen solmsi*). Together with the result from *C. chilonis*, this indicates that the threonine dehydratase gene might have been lost in the common ancestor of Parasitoida. In addition, we observed that this gene was also lost in a eusocial wasp *Polistes canadensis* and three ants, but retained in bees and nine other ants, suggesting independent gene losses in the infraorder Aculeata. Similarly, the two arginine biosynthetic pathway genes argininosuccinate synthase and argininosuccinate lyase were lost in 21 and 23 arthropods, respectively (19 species lost both genes), due to a number of independent gene losses in different lineages during Arthropoda evolution (Additional file 1: Table S6). We found that these two genes were also lost in a closely related species of *C. chilonis* (*M. demolitor*) but present in another parasitoid wasp *N. vitripennis*, implying that the gene losses might have occurred in the common ancestor of *C. chilonis* and *M. demolitor*. Interestingly, in contrast to the losses of three pathway genes during Arthropoda evolution, the presence of the cysteine synthase in *C. suppressalis* is probably due to a gene gain event, because we found this gene in only 10 of 72 arthropods (8 Lepidopterans, 1 Collembolan, and 1 Arachnidan) (Additional file 1: Table S6). All lepidopterans we examined have this gene, suggesting an ancient gene gain before the divergence of Lepidoptera. Additional phylogenetic analysis of a large number of species from different taxa also supported this conclusion (Additional file 2: Fig. S2). Overall, pathway reconstruction analysis indicated that the parasitoid *C. chilonis* and its host *C. suppressalis* both lost the ability to synthesize nine amino acids (ASL-AA), and this represents the ancestral states. Moreover, *C. chilonis* further lost the ability to synthesize arginine due to two pathway gene losses during Hymenoptera evolution. Although the amino acid biosynthetic pathways are largely conserved, the loss and gain of several pathways are ongoing.

### In vitro verification of the requirements for different amino acids

**Fig. 3:**
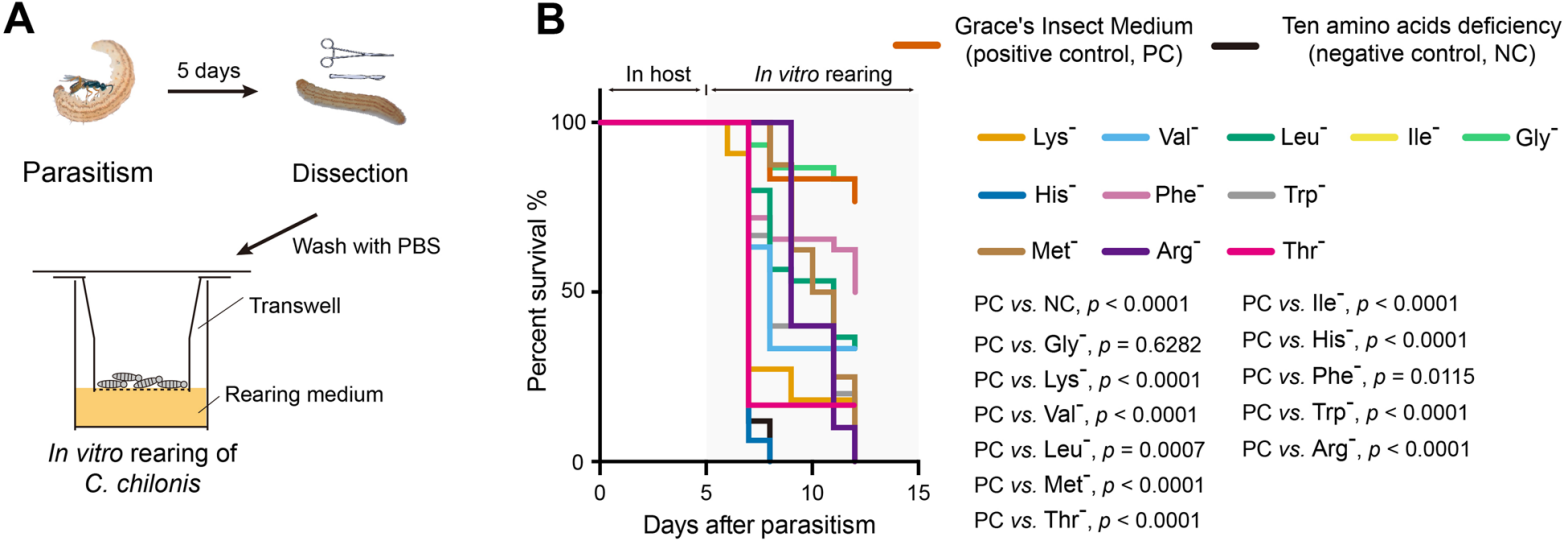
Rearing of *C. chilonis* with different rearing media in vitro. **A** In vitro rearing of *C. chilonis*. Five days after parasitism, larvae were put on the membrane of a Transwell chamber; then, the Transwell was placed in the well containing 250 μl of *C. chilonis* rearing medium so that the wasp larvae could reach the nutrients. **B** Survival rates of *C. chilonis* larvae developed on 13 different rearing media. Positive control: Grace’s Insect Medium, containing 20 amino acids (*n* = 30); negative control uses positive control medium minus the ten amino acids that *C. chilonis* cannot synthesize, ASL-AA, Arg, *n* = 30; single amino acid deficiencies use control media minus only one amino acid, indicated by “-” superscript, e.g., Gly deficiency (Gly^-^) indicates excluding glycine only. The Gehan-Breslow-Wilcoxon test was used for survival rate statistical analyses, and the Benjamini-Hochberg method was used for multiple testing correction. The statistical results of pairwise group comparisons are indicated

To study the effect of loss of ability to synthesize certain amino acids on larval development, we reared 5-day-old *C. chilonis* larvae in vitro in a chemically defined medium (Fig. 3; see the “11” section). Grace’s Insect Medium, which contains 20 amino acids, was used as a positive control, and the medium without the ten essential amino acids (ASL-AA and arginine) was used as a negative control. Ten different media, each lacking a single amino acid, were used to test the requirement for these essential amino acids. The results indicated that the absence of any of the ten essential amino acids led to the developmental arrest of parasitoid larvae, demonstrating that *C. chilonis* larvae cannot survive without these ten amino acids. However, they could survive in the medium without glycine, presumably because of their ability to synthesize it (based on the pathway analysis, Fig. 2). Our results are consistent with those of previous studies and explain why parasitoid wasps cannot survive on chemically defined media lacking one or more of these critical nutrients [33–35].

### Parasitism alters the amino acid contents of host hemolymph in different ways

**Fig. 4:**
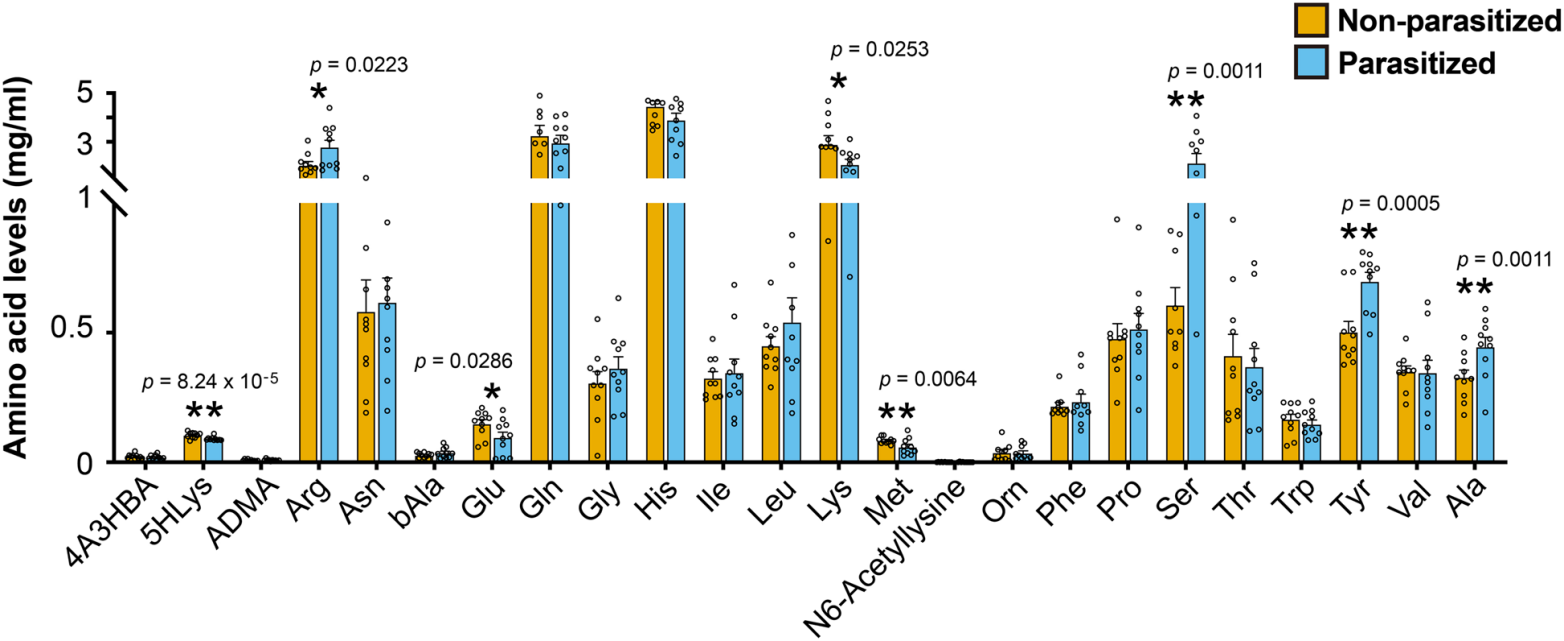
Parasitism by *C. chilonis* influences the free amino acid levels in host hemolymph. Free amino acid levels in host hemolymph were changed after parasitism. UPLC-MS/MS analysis was used. Host hemolymph was collected 3 days after parasitism. The detection for each treatment was repeated 10 times (*n* = 10). Student’s *t*-test was used for statistical analysis of amino acid changes. *p < 0.05, **p < 0.01

Pathway analyses and feeding assays showed that *C. chilonis* has lost the ability to synthesize ten essential amino acids. Next, we asked how parasitism alters the amino acid contents in host hemolymph. To this end, a comparative metabolomics analysis was conducted. In non-parasitized *C. suppressalis* larvae, all ten essential amino acids were present in the hemolymph (Additional file 1: Table S7). Since the biosynthetic pathways of nine amino acids (not including arginine) were lost in *C. suppressalis*, we speculated that the host *C. suppressalis* obtain these amino acids from its diet because symbiotic bacteria are not found in this insect. Histidine was the most abundant at a concentration of 4.455 mg/ml in the hemolymph, followed by glutamine at a concentration of 3.262 mg/ml (Fig. 4). In the parasitized larvae, the amounts of four amino acids (arginine, serine, tyrosine, and alanine) were significantly increased, and four amino acids (5-hydroxylysine, glutamic acid, methionine, and lysine) were significantly decreased after the first 72 h of parasitism (*p* < 0.05, Student’s *t*-test). Among the ten essential amino acids, at the first 72 h after parasitism, lysine and methionine were significantly decreased in abundance while arginine was significantly increased in the host hemolymph after the first 72 h of parasitism (Fig. 4). The levels of the other seven amino acids (threonine, tryptophan, phenylalanine, isoleucine, leucine, valine, and histidine) remained unchanged (Fig. 4). In summary, parasitism by *C. chilonis* changes the amino acid levels in the host hemolymph at the first 72 h after parasitism. It should be noted that measurements at a single time point cannot reveal the whole landscape of amino acid changes in the host. The decrease in amino acid contents is likely due to nutrient absorption by the parasitoid larvae [1]. However, the mechanism underlying the increase of amino acid content in the host hemolymph after parasitism is still unknown.

### Gene expression changes in the host *C. suppressalis* induced by parasitism

We proposed three hypotheses that may explain the increases of amino acid content in host hemolymph after parasitism. The first is that the parasitoid activates the biosynthesis of amino acids in the host, the second is that the parasitoid inhibits the consumption of amino acids by the host, and the third is that the parasitoid stimulates the protein degradation in the host. To test these hypotheses, we performed transcriptome sequencing of unparasitized and parasitized hosts at 24 h, 48 h, and 72 h after parasitism. The differentially expressed genes were then analyzed.

**Fig. 5:**
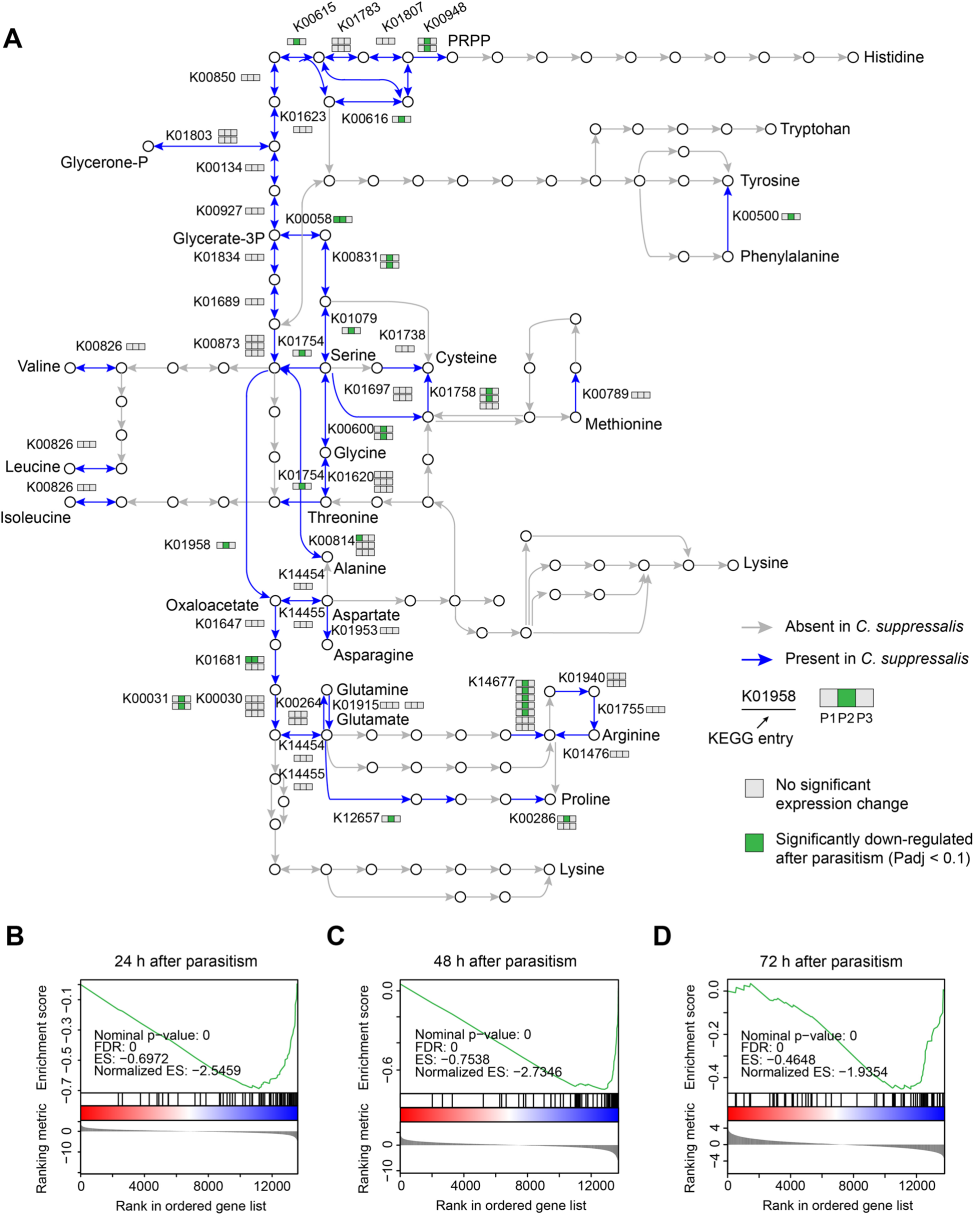
Parasitism by *C. chilonis* suppresses the host’s amino acid biosynthetic pathways within 72 h after parasitism. **A** The amino acid biosynthetic pathways were modified from Fig. 2. The pathway genes in gray are lost in the genome of *C. suppressalis*. The pathway genes in green are present in the genome of *C. suppressalis*. Gene expression changes at 24 h (P1), 48 h (P2), and 72 h (P3) after parasitism were shown in three squares with different colors (gray, no significant expression change; green, significantly downregulated after parasitism, padj < 0.1). Gene set enrichment analyses (GSEA) showed that the genes in the amino acid biosynthetic pathways of *C. suppressalis* were largely downregulated at 24 h (**B**), 48 h (**C**), and 72 h (**D**) after parasitism. The top portion of the GSEA plot shows the running enrichment score for the gene set as the analysis walks down the ranked list. The middle portion of the GSEA plot reflects the positions of the members of the gene set in the ranked gene list. The bottom portion of the GSEA plot displays the value of the ranking metric along with the list. Three independent biological replicates of each sample for gene expression analyses were conducted (*n* = 3). Expression source data are included in Additional file 1: Table S8

First, we examined the expression changes of genes in the amino acid biosynthetic pathways. Gene set enrichment analysis (GSEA) was used to identify the changes in the expression of a specific gene set. The results showed that the expression of amino acid synthetic pathway genes decreased within 72 h after parasitism (Fig. 5), suggesting that parasitism suppressed amino acid synthesis in the host. Specifically, 25 amino acid synthetic pathway genes were significantly downregulated at 48 h after parasitism (padj < 0.1), which is much greater than the number at 24 h or 72 h after parasitism (Fig. 5A and Additional file 1: Table S8). Among these 25 genes, 15 belong to the synthetic pathways of non-essential amino acids. Since there were not any significantly upregulated genes within 72 h after parasitism, we rejected the first hypothesis. In other words, parasitism by *C. chilonis* had an overall inhibitory effect on amino acid synthesis in the host within 72 h after parasitism, which did not contribute to the increase in the amino acid content in the host hemolymph.

**Fig. 6:**
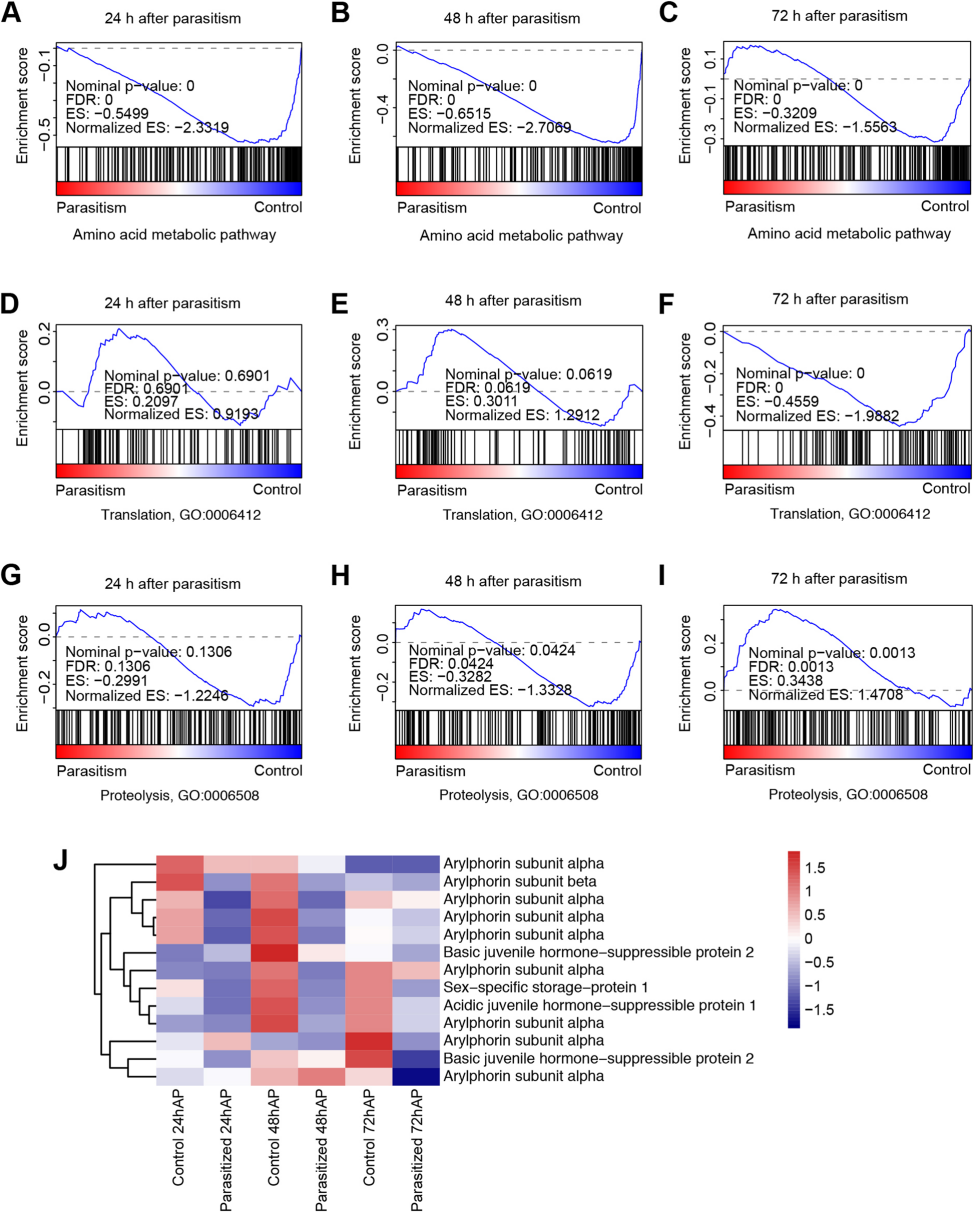
Parasitism by *C. chilonis* inhibits host amino acid utilization and activates host protein degradation within 72 h after parasitism. Gene set enrichment analyses (GSEA) showed that the genes in the amino acid metabolic pathways of *C. suppressalis* were largely downregulated at 24 h (**A**), 48 h (**B**), and 72 h (**C**) after parasitism. GSEA also revealed various expression changes of translation-related genes (GO: 0006412) at 24 h (**D**) and 48 h (**E**) after parasitism, and a significantly downregulated effect at 72 h (**F**) after parasitism. In addition, a significantly upregulated pattern of the host proteolysis-related gene set (GO: 0006508) at 72 h (**I**) after parasitism was found, but not at 24 h (**G**) or 48 h (**H**) after parasitism. Moreover, the expression of host storage protein genes was significantly inhibited within 48 h after parasitism (**J**). But we did not obverse statistical significantly differential gene expression at 72 h after parasitism. The gene expressions between parasitized hosts and controls at 24 h after parasitism (24hAP), 48 h after parasitism (48hAP), and 72 h after parasitism (72hAP) were compared respectively. The mean expression level (FPKM) of each treatment was used for heatmap plotting. Three independent biological replicates of each sample for gene expression analyses were conducted (*n* = 3). Expression source data are included in Additional file 1: Tables S9-S21

Next, we tested whether parasitism downregulated the consumption of amino acids in the host. GSEA revealed that the expression of 205 genes in the amino acid metabolic pathways in the host was significantly inhibited within 72 h after parasitism (*p* < 0.001, Fig. 6A–C, Additional file 1: Tables S9-S18). These results indicate that parasitism might cause the accumulation of amino acids in host hemolymph by downregulating amino acid metabolism. However, this overall inhibitory pattern is not consistent with the temporal increase in the concentrations of only four amino acids (arginine, serine, tyrosine, and alanine) after parasitism. In addition, we asked whether parasitism influences the process of protein translation in the host. GSEA showed various expression changes of 104 predicted translation-related genes (GO: 0006412) at 24 h and 48 h after parasitism, as well as a significant downregulation at 72 h after parasitism (*p* < 0.001, Fig. 6D–F, Additional file 1: Table S19). Taken together, our observations showed that parasitism may induce the increase in amino acid content in host hemolymph by inhibiting the utilization of amino acids by processes such as amino acid metabolism and translation, which supports the second hypothesis.

We next investigated how parasitism influences the proteolysis process in the host. The results showed a clear upregulation of the proteolysis-related gene set (174 genes, GO: 0006508) at 72 h after parasitism (*p* < 0.001, Fig. 6I, Additional file 1: Table S20). Given the important function of insect storage proteins in supplying amino acids for insect development, we identified insect storage protein genes in the host genome and examined their expression changes after parasitism. In total, 13 insect storage protein genes were annotated, including nine arylphorin genes, one sex-specific storage protein gene, one acidic juvenile hormone-suppressible protein gene, and two basic juvenile hormone-suppressible protein genes. Differential expression analysis revealed that three arylphorin genes and one sex-specific storage protein gene were significantly downregulated in the host at 24 h and 48 h after parasitism, and an additional arylphorin gene and one acidic juvenile hormone-suppressible protein gene were significantly downregulated at 48 h after parasitism (log_2_(fold change) < − 2 and *p* < 0.05, Fig. 6J and Additional file 1: Table S21). However, we did not detect any statistically significant change in the gene expression at 72 h after parasitism, possibly due to the poor repeatability. Taken together, these results likely support the third hypothesis we proposed and suggest that parasitism may activate the proteolysis process in the host and cause the degradation of insect storage proteins. This in turn releases free amino acids into the hemolymph, providing an alternative explanation for why parasitism increases the amino acid content in the host hemolymph.

## Discussion

Here, we integrate genomic, transcriptomic, and metabolomic data to shed light on the molecular mechanism by which parasitoid wasps exploit host amino acids. We first presented genome-level evidence that *C. chilonis* has lost the capability for de novo biosynthesis of ten essential amino acids (ASL-AA and arginine). This was confirmed in in vitro experiments in which *C. chilonis* larvae were reared in media lacking one or more specific amino acids. Metabolomic analysis revealed that all ten amino acids were available in the host hemolymph and that the amino acid contents in hemolymph were altered in parasitized hosts within the first 72 h after parasitism. Meanwhile, the transcriptomic analysis showed that, on the one hand, parasitism significantly inhibited the expression of host genes involved in the biological processes related to amino acid utilizations, such as amino acid metabolism and translation; on the other hand, parasitism significantly stimulated protein degradation processes in the host. The differential expression of these genes partly explains the increase in amino acid content in host hemolymph after parasitism. Overall, the results have substantially answered the question of how the parasitoid wasps exploit host amino acids. The amino acid levels in host hemolymph and the expression of several amino acid-related genes in the host were temporally altered after parasitism by parasitoid wasps. Further studies are needed to investigate the direct connection between the effectors of parasitoid wasps (e.g., venom and PDVs) and the changes in amino acid concentrations in the host.

Pathway analysis showed that the loss of the capability to synthesize several amino acids in *C. chilonis* was caused by the loss of one or several key pathway genes, and this loss was also observed in the host *C. suppressalis*. Comparative pathway analysis revealed that the amino acid synthesis gene repertoire is very similar between *C. chilonis* (Hymenoptera) and *C. suppressalis* (Lepidoptera). This finding is consistent with our previous understanding that essential amino acid biosynthesis was lost in the ancestor of animals [25]. The host *C. suppressalis* genome has all the pathway genes of *C. chilonis*, although the gene copy numbers are variable. In contrast, four amino acid synthetic pathway genes existing in the *C. suppressalis* genome were lost in the *C. chilonis* genome. We investigated these repertoire differences in a phylogenetic context through the extended sampling of arthropods. The results indicated that three of the differences were caused by gene losses in *C. chilonis* and another was caused by gene gain in *C. suppressalis*. This suggests that pathway gene losses and gains occurred during the evolution of arthropods, although the amino acid synthetic pathways are largely conserved. Studies of more arthropod species are required to uncover the evolution of amino acid synthesis pathways.

Based on the pathway completeness analysis, both parasitoid *C. chilonis* and its host *C. suppressalis* lost the ability to synthesize nine amino acids (ASL-AA). Furthermore, *C. chilonis* lost the ability to synthesize arginine due to the loss of two genes, argininosuccinate synthase and argininosuccinate lyase. Further investigation of 72 arthropods revealed that these two genes were independently lost in many other species from different lineages, such as bee, ant, aphid, bed bug, whitefly, fruit fly, louse, mite, tick, and springtail (Additional file 1: Table S6). This result suggests that gene loss in the arginine biosynthesis pathway is relatively prevalent among arthropods. However, these gene losses may be due to the totally different factors in different organisms. The gene losses and the loss of ability to synthesize specific amino acids in some arthropods may reflect their specialized diets. For example, bees feed on amino acid-rich pollen, and ants have a wide range of food sources [36, 37]. Another possibility is that symbiotic bacteria can provide essential nutrients to host insects, as reported in *Cephalotes* ants [38], and almost all phloem-sapping insects such as aphids [39, 40]. In parasitoid wasps, based on pathway analysis, we found that two braconid wasps, *C. chilonis* and *M. demolitor*, have lost the ability to synthesize arginine. The loss of arginine biosynthesis in parasitoid wasps may be explained by their amino acid-rich diet combined with the capability of parasitoids to manipulate the nutritional quality of hosts through venoms and other means. However, the biosynthetic pathway from aspartate to arginine is complete in another parasitoid wasp, *N. vitripennis*.

Previous studies have reported that parasitism can regulate the host metabolic system and release nutrients into hemolymph to increase nutritional suitability for parasitoid larvae through venoms, teratocytes, and parasitoid larval feeding [1, 8–10, 14, 15, 41–45]. A study of the same parasitoid-host system suggested that parasitism likely first increases amino acid levels in host hemolymph, then newly hatched parasitoids probably begin to consume these amino acids during development [46]. Metabolomic analysis showed that parasitism by *C. chilonis* significantly changed the levels of various amino acids in host hemolymph (Fig. 4). For the ten essential amino acids, the concentrations of lysine, methionine, and arginine were found to change significantly in host hemolymph after parasitism. It should be noted that we only monitored the amino acid content change in host hemolymph at a single time point after parasitism. Therefore, it is impossible to determine how the amino acid content changes over time. To understand the landscape of the amino acid changes in host hemolymph, more intensive sampling is required. There are various reasons for the changes in amino acid contents. For example, parasitoid larvae may directly absorb amino acids as nutrition, thus resulting in the decrease of amino acid content [1, 45]. However, whether parasitoid larvae selectively absorb specific amino acids is still unknown. In addition, some amino acids can be used as precursors to produce other compounds, which may also cause a decrease in amino acid contents in the host. For instance, glutamate is a precursor of *gamma*-aminobutyric acid, a chief inhibitory neurotransmitter [47, 48], and tyrosine is a precursor of dopamine, another key neurotransmitter in insects [49]. Moreover, venom and/or other effectors produced by parasitoids (i.e., PDVs, teratocytes) and/or by larvae feeding could presumably increase amino acid levels in host hemolymph; and this hypothesis has been partly verified in a previous study [1, 8].

Transcriptomic analysis showed an obvious downregulation of genes in the amino acid biosynthetic pathways of the host after parasitism. The inhibition of host amino acid biosynthesis may affect a range of biological processes in the host and eventually weaken the host’s defense against parasitoid wasps. Meanwhile, the amino acid utilization of the host was inhibited, and the protein degradation of the host was activated, which may partly explain the amino acid level increase in host hemolymph after parasitism. Though we found that the contents of four amino acids were increased, the mechanism remains unknown. Moreover, it remains unclear which effector regulates the gene expression changes in the parasitized host.

Many parasitoid wasps are important natural enemies of agriculture and forestry pests, and they have been widely used as biological control agents [50–52]. The use of artificial diets for mass rearing of parasitoids is an important part of efforts to increase their utility and cost-effectiveness for augmentative biological control. Here, we provide evidence for the necessary amino acids that need to be added as supplements to artificial diets.

## Conclusions

We present a genome of the parasitoid wasp *C. chilonis* to understand the genetic basis of trait loss in amino acid biosynthesis and amino acid resource exploitation. Amino acid biosynthetic pathway analyses and feeding experiments indicated that *C. chilonis* lost the ability to synthesize ten amino acids. And the biosynthetic pathways of nine of them have been disrupted in the common ancestor of animals. Comparative genomics analyses showed that the loss of arginine synthesis is common in arthropods, including in *C. chilonis*. Further transcriptomic and metabolomic studies illustrated that *C. chilonis* inhibit amino acid utilization and activate protein degradation in the host, likely resulting in the increase of amino acid content in host hemolymph. Altogether, our discoveries lay a blueprint for future research on the mechanisms underlying the exploitation of host nutrition by parasitoid wasps, and this knowledge can specifically be used to design parasitoid artificial diets which potentially benefit mass rearing of parasitoids for pest control.

## Methods

### Insects

The parasitoid wasp *C. chilonis* and its host *C. suppressalis* were collected from fields in the experimental farmland of Zhejiang University, Hangzhou, China, in 2012 and reared under laboratory conditions as previously described [26, 27].

### Genome sequencing, assembly, and annotation

We used Illumina HiSeq 2000 and Pacbio platforms to sequence the genome of *C. chilonis*. To minimize the potential effects of heterozygosity in genome assembly, the sequencing strain had six generations of sib mating. DNA was extracted from 300 haploid third-instar male wasp larvae. Sequencing libraries with insert sizes of 250 bp, 2 kb, 5 kb, and 10 kb for paired-end reads were constructed. In total, we generated about 64.44 Gb of Illumina reads and 7.63 Gb of Pacbio reads (~ 380 X coverage) (Additional file 1: Table S1). Canu version 1.4 [53] was used for de novo assembly with Pacbio long-read data, and the SSPACE software (version 3) [54] was applied for scaffolding with pair-end short-read data. The PILON software [55] was used for error correction with Illumina data. The final assembly yielded 189 Mb of the reference genomic sequence with a scaffold N50 of 2.2 Mb (Table 1). BUSCO version 5 [28] was used to assess the completeness of the assembly.

To further obtain a chromosome-level scaffold assembly of *C. chilonis*, we next used the Hi-C technique to detect chromosomal contact information. Hi-C libraries were constructed using 50 male pupae as input, and the Hi-C library generation standard protocol for parasitoid wasps was previously described [3], including cross-linking, chromatin digestion, marking of DNA ends with biotin-14-dCTP, ligation and purification, shearing, and biotin pull down. Then, the Hi-C library was then sequenced on the Illumina HiSeq X Ten platform (150 paired-end). For Hi-C scaffolding, Hi-C reads were mapped to the genome by BWA version 0.6.2 [56] with default parameters. Unmapped paired-end reads and multiple mapped reads were filtered by samtools version 1.9 [57]. LACHESIS (https://github. com/shendurelab/LACHESIS) was further applied to order and orient the clustered contigs [58]. Assembly errors were manually corrected in JucieBox version 1.8.8 [59]. Finally, we obtained the chromosomal-level assembly with ten chromosomes, chromosomal lengths from 27.2 to 10.6 Mb containing 91.9% of the total sequence (Additional file 1: Table S3).

We then used the RepeatModeler and RepeatMasker software (version 4.0.5) [60] to identify repeat sequences in the *C. chilonis* genome with default parameters. Next, we used the OMIGA genome annotation pipeline [29] to annotate the coding sequences in the *C. chilonis* genome by integrating evidence from de novo predictions, homolog mapping, and gene expressions. Three gene prediction programs, including Augustus version 3.1 [61], SNAP (version 2006-07-28) [62], and GeneMark-ET (Suite 4.21) [63] were used for de novo gene prediction. For homolog searching, sequences of homologous proteins from the NCBI invertebrate RefSeq were used. Additionally, the six transcriptomic data from different development stages were mapped to the genome using HISAT2 version 2.2.1 [64], and StringTie version 2.1.0 [65] was used to assemble the transcripts to the gene models. All gene sequences predicted from the above three approaches were combined by MAKER into a weighted and non-redundant consensus of gene structures. All the MAKER parameters were default settings. In total, OMIGA identified 14,142 protein-coding genes in the *C. chilonis* genome.

### Transcriptome sequencing and analysis

Transcriptomic libraries were prepared from early larvae (3 days after parasitism), later larvae (9 days after parasitism), male pupae, female pupae, male adults, and female adults of *C. chilonis*. Additionally, to examine the host gene expression changes after parasitism, 18 RNA-seq libraries were prepared from the unparasitized and parasitized hosts (third-instar larvae) 1 day, 2 days, and 3 days after parasitism. Each sample had three independent biological replicates. The Illumina HiSeq X Ten platform was used for transcriptome sequencing (insert size of 250 bp). The clean reads were mapped to the genome assembly using HISAT2 version 2.2.1 and assembled into transcripts by StringTie version 2.1.0. RSEM version 1.3.0 [66] used for calculating the gene expression levels. Differentially expressed genes were identified using DESeq2 (version 1.30.1) [67]. Benjamini-Hochberg correction was used to adjust *p* values for multiple testing (FDR adjusted). Gene set enrichment analysis (GSEA version 4.1.0) [68] was used to determine whether a priori defined set of genes (based on KEGG pathways and Gene Ontology Annotations) shows statistically significant gene expression differences between unparasitized and parasitized hosts.

### Phylogenetic analysis

All the 2179 single-copy homologous genes identified by the OrthoFinder version.2.3.5 [69] among species (*C. chilonis*, *Cotasia flavipes*, *Cotesia sesamiae*, *Cotesia vestalis*, *Cotesia glomrata*, *Cotesia rubecula*, *Cotesia congregata*, *Microplitis demolitor*, *Chelonus insularis*, *Macrocentrus cingulum*, *Habrobracon hebetor*, *Diadromus collaris*, *Pteromalus puparum*) were aligned and trimmed using MAFFT version 7 and trimAl version 1.2 [70, 71] and concatenated into supergenes for phylogenetic relationship analyses. Maximum likelihood-based phylogenetic analysis was conducted using IQ-TREE version 2.1.2 [72], and values of statistical support were obtained from 1000 replicates of bootstrap analysis.

### Amino acid synthetic pathway reconstruction and analysis

We applied a pathway annotation pipeline to identify the amino acid synthetic pathway genes in the *C. chilonis* genome and its host *C. suppressalis* genome. First, to minimize the impact on this analysis of genome contamination with bacterial sequences in the assemblies, we used a method to detect bacterial-contaminated scaffolds in these two genomes [3, 73]. In total, 804 bacterial scaffolds with an average length of 6345 bp were found in the *C. suppressalis* genome, and only one bacterial scaffold in 11,968 bp was found in the *C. chilonis* genome. These bacterial scaffolds were subsequently removed from the analysis. Then, a pathway annotation tool BlastKOALA v2.2 [74] was used to identify the genes in the amino acid biosynthetic pathway (pathway name: 01230 Biosynthesis of amino acids). To avoid missing genes during annotation, we used TBLASTN to scan genes in genome assembly with Evalue < 10^−5^ and coverage above 75%. All annotated pathway genes were manually checked by BLASTP and Pfam online tools, and these genes were further verified by checking their complete open reading frames. The pathway disruptions and gene losses were identified using a KEGG online tool, KEGG Mapper (https://www.kegg.jp/kegg/mapper.html). The amino acid biosynthetic capability was evaluated in terms of metabolic pathway completeness. The loss of synthesis capability to a particular amino acid occurs when all currently known synthetic pathways for the amino acid are disrupted.

### Rearing *C. chilonis* larvae in vitro

Based on the protocols for rearing other parasitoid wasps [33, 34], the chemically defined rearing media were prepared in our laboratory following the composition of Grace’s Insect Medium (Thermo Fisher, catalog number: 11605; see detail components and concentrations in Additional file 1: Table S22). All chemicals were obtained from Sigma Chemical Company (Shanghai, China). To verify the requirements for the ten different amino acids of *C. chilonis*, we in vitro reared wasp larvae in the different mediums. The Grace’s Insect Medium was used as a positive control, and the baseline medium which deleted ten amino acids (ASL-AA and arginine) that *C. chilonis* cannot synthesize was used as a negative control. Eleven other mediums were formulated by deleting only one amino acid (including ten amino acids which *C. chilonis* cannot synthesize and one amino acid (glycine) which *C. chilonis* can synthesize). The deletion was accompanied by proportional increases in the quantity of all the remaining amino acids to maintain a constant amino acid level through adding a single amino acid, namely, glutamate, as described previously [33, 34]. Each artificial rearing medium was then sterilized by passing through a 0.22-mm filter (Merck Millipore Ltd.; Tullagreen, Carrigtwohill, Co. Cork, IRL). Each solution was then stored at − 20 °C until use.

The protocol for rearing parasitoid wasps in vitro was based on the methods used for *N. vitripennis* [75, 76]. For each tested rearing medium, the larvae of *C. chilonis* were collected by dissecting the parasitized *C. suppressalis* larvae, which were cleaned with 70% ethanol for surface sterilization 5 days after parasitism. Ten wasp larvae were transferred onto a 3-μm pore Transwell polyester membrane (Costar; Corning Incorporated, Corning, NY, USA) after washing with 1× phosphate buffer saline (PBS) three times. Then, the Transwell insert was transferred to a well with 250 μl of rearing medium in a 24 well plate. All plates were stored in a sterile Tupperware box at 27 ± 1 °C for the duration of the experiment. To confirm whether the larvae were alive, body movement, gut movement, and body color were considered as criteria. The rearing experiments were replicated three times. Photos were also taken every day, and the larvae body lengths were measured using the ImageJ software (version 1.47).

### Metabolomics analysis for free amino acids in host hemolymph

Third-instar larvae of parasitized and non-parasitized *C. suppressalis* were surface-sterilized with 75% ethanol. Their prolegs were then cut with a pair of scissors and 30 μl of hemolymph was collected using micropipette tips and transferred into a 1.5-ml Eppendorf tube containing 10 μl saturated α-phenylthiourea (PTU). After a brief centrifugation, 20 μl supernatant of hemolymph without hemocytes was collected and mixed with 80 μl of pre-cooled methanol. The mixture was vortexed for 1 min. After overnight incubation at 4 °C, the sample was centrifuged at 14,000*g* for 15 min at 4 °C. The resulting supernatant (10 μl) was diluted 20-fold with 50% aqueous acetonitrile and subsequently mixed with an equal volume of internal standard solution (ISs) (100 ng/ml in 50% aqueous acetonitrile) prior to UPLC-MS/MS analysis with 1 μl of injection volume.

The UPLC-MS/MS analysis was performed on a Waters Acquity UPLC System (Waters, Milford, MA) coupled to a Triple Quad™ 5500 tandem mass spectrometer (AB Sciex, Framingham, MA), and 3 μl of each sample or calibration curve sample was injected onto a Waters BEH Amide column (100 mm × 2.1 mm, 1.7 μm) at a flow rate of 0.4 ml/min. The mobile phase consisted of (A) water with 10 mM ammonium formate and 0.2% formic acid and (B) acetonitrile with 2 mM ammonium formate and 0.2% formic acid. The chromatographic separation was conducted with a gradient elution program as follows: 0 min, 90% B; 0.5 min, 90% B; 5.5 min, 75% B; 6.5 min, 50% B; 7.5 min, 50% B; 7.51 min, 90% B; 10 min, 90% B. The column temperature was maintained at 40 °C.

The samples eluted from the column were ionized in an electrospray ionization source in positive mode (ESI+). Source temperature, 550°C; curtain gas (CUR), 35 psi; ion source gas 1 (GS1), 50 psi; ion source gas 2 (GS2), 50 psi; collision gas (CAD), 8 psi; ion spray voltage (IS), 5500 V; entrance potential (EP), 10 V; and collision cell exits potential (CXP1), 10 V. The scheduled multiple reaction monitoring (sMRM) was used to acquire data in optimized MRM transition (precursor > product), declustering potential (DP), and collision energy (CE) as shown in Additional file 1: Table S23. The test samples and standard curve samples were analyzed simultaneously. The AB Sciex Analyst software (version 1.5.2) was used to control instruments and acquire data.

## Supplementary Information

**Additional file 1: Table S1.** Genome sequencing data of *C. chilonis*. **Table S2**. Hi-C sequencing data of *C. chilonis*. **Table S3.** Hi-C scaffolding and assembled chromosomes (superscaffolds) of *C. chilonis*. **Table S4.** Classification of repeat sequences identified in the *C. chilonis* genome. **Table S5.** Amino acid biosynthetic pathway disruptions in *C. chilonis* and *C. suppressalis*. **Table S6.** Numbers of the four amino acid biosynthetic pathway genes in 72 arthropods from KEGG pathway database. **Table S7.** The concentration changes of each amino acids in host hemolymph after parasitism, this data is used for plotting Fig. 4. **Table S8.** Expression changes of amino acid synthetic pathway genes within three days after parasitism (FPKM values). **Table S9.** Expression changes of alanine, aspartate and glutamate metabolic pathway genes within three days after parasitism (FPKM values). **Table S10.** Expression changes of glycine, serine and threonine metabolic pathway genes within three days after parasitism (FPKM values). **Table S11.** Expression changes of cysteine and methionine metabolic pathway genes within three days after parasitism (FPKM values). **Table S12.** Expression changes of valine, leucine and isoleucine metabolic pathway genes within three days after parasitism (FPKM values). **Table S13.** Expression changes of lysine metabolic pathway genes within three days after parasitism (FPKM values). **Table S14.** Expression changes of arginine and proline metabolic pathway genes within three days after parasitism (FPKM values). **Table S15.** Expression changes of histidine metabolic pathway genes within three days after parasitism (FPKM values). **Table S16.** Expression changes of tyrosine metabolic pathway genes within three days after parasitism (FPKM values). **Table S17.** Expression changes of phenylalanine metabolic pathway genes within three days after parasitism (FPKM values). **Table S18.** Expression changes of tryptophan metabolic pathway genes within three days after parasitism (FPKM values). **Table S19.** Expression changes of translation-related genes (GO: 0006412) within three days after parasitism (FPKM values). **Table S20.** Expression changes of proteolysis-related gene set (GO: 0006508) within three days after parasitism (FPKM values). **Table S21.** Expression changes of insect storage protein genes within three days after parasitism (FPKM values). **Table S22.** Composition of Grace’s Insect Medium, according to Thermo Fisher, catalog number: 11605. **Table S23.** Parameters for quantification of 28 amino acids by UPLC-MS/MS.

**Additional file 2: Fig. S1.** Heatmap of the all-by-all interactions among 10 chromosomes of *C. chilonis*. **Fig. S2.** Phylogenetic analysis of cysteine synthase genes, suggesting an ancient gene gain event in Lepidoptera.

## Data Availability

All sequence data of the *C. chilonis* genome project have been deposited in GenBank under the accession code RJVT00000000 [77]. In addition, all the data in this paper have been deposited in the InsectBase 2.0 [78] (InsectBase ID: IBG_00206 [79]). Transcriptome data have been deposited in SRA under SRX14365338-SRX14365355 [80]. Source data of gene expressions supporting the analyses in this study are included in Additional file 1: Tables S8-S21. All data generated or analyzed during this study are included in this published article, its supplementary information files, and publicly available repositories.
