# Supplementary material for: Genome of the parasitoid wasp Cotesia chilonis sheds light on amino acid resource exploitation
Source: BMC Biol. 2022 May 24;20:118. doi: 10.1186/s12915-022-01313-3 (PMC9128236; doi:10.1186/s12915-022-01313-3)
Supplement: Supplementary file 2 — Additional file 2: Fig. S1. Heatmap of the all-by-all interactions among 10 chromosomes of C. chilonis. Fig. S2. Phylogenetic analysis of cysteine synthase genes, suggesting an ancient gene gain event in Lepidoptera. [file 12915_2022_1313_MOESM2_ESM.pdf]

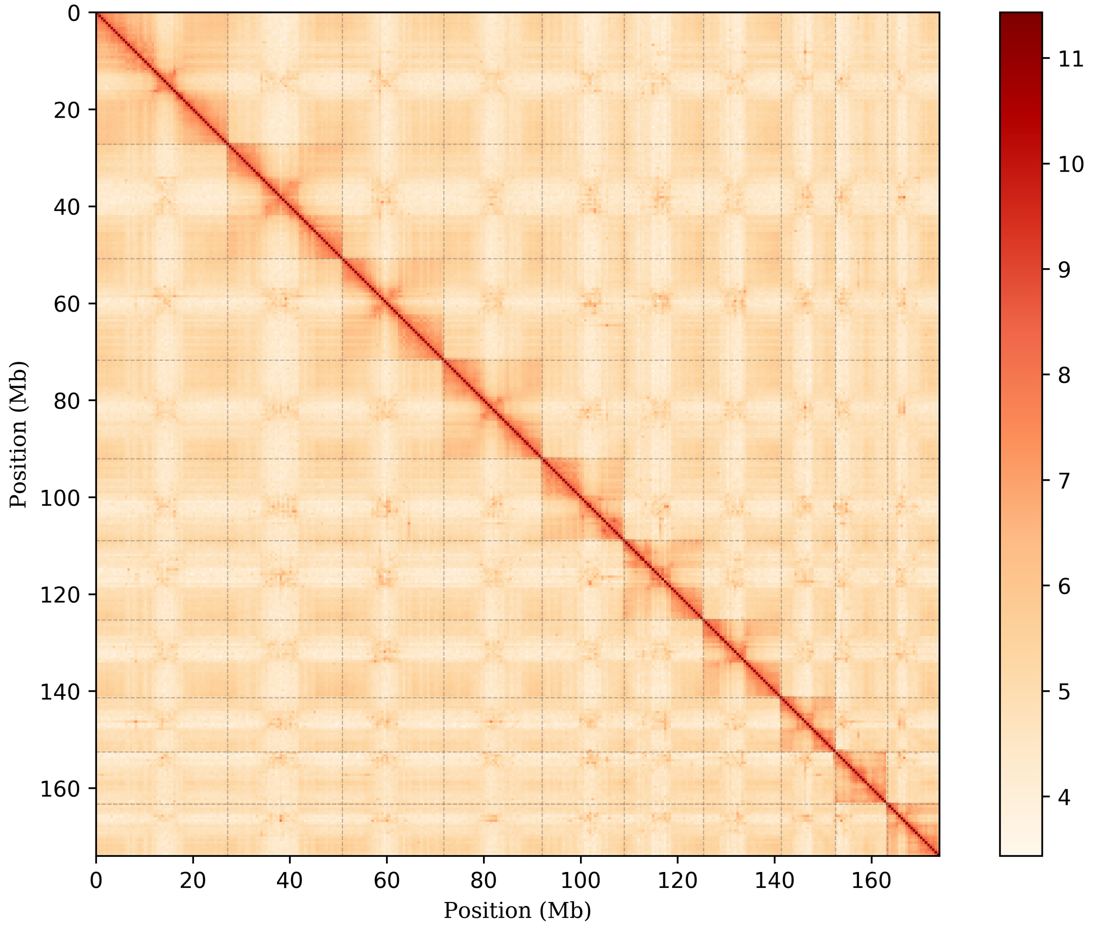

Supplementary Figure 1. Heatmap of the all-by-all interactions among 10 chromosomes of *C. chilonis*.

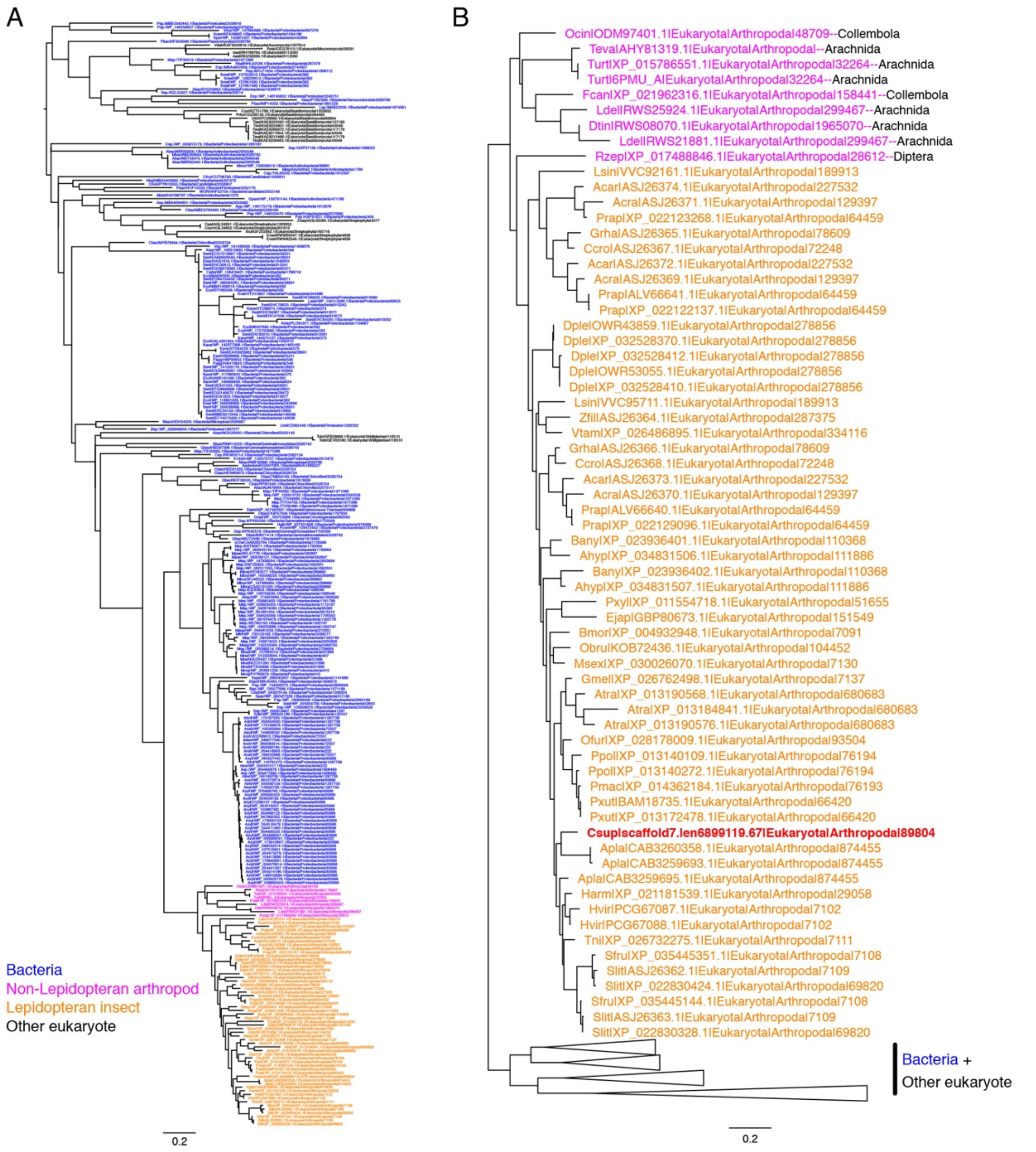

Supplementary Figure 2. Phylogenetic analysis of cysteine synthase genes, suggesting an ancient gene gain event in Lepidoptera. (A) Phylogenetic tree of cysteine synthase genes. Genes from bacteria, non-Lepidopteran arthropods, lepidopteran insects, and other eukaryote species are shown by blue, pink, orange, and black, respectively. In addition, the clade containing arthropods is shown in (B). The gene from *C. suppressalis* is shown in red.
